# Context-independent expression of spatial code in hippocampus

**DOI:** 10.1038/s41598-022-25006-7

**Published:** 2022-12-01

**Authors:** S. Kapl, F. Tichanek, F. Zitricky, K. Jezek

**Affiliations:** Faculty of Medicine in Pilsen, Charles University, 32300 Pilsen, Czech Republic

**Keywords:** Hippocampus, Long-term memory, Neurophysiology, Cognitive neuroscience

## Abstract

The hippocampus plays a crucial role in the formation and retrieval of spatial memory across mammals and episodic memory in humans. Episodic and spatial memories can be retrieved irrespective of the subject’s awake behavioral state and independently of its actual spatial context. However, the nature of hippocampal network activity during such out-context retrieval has not been described so far. Theoretically, context-independent spatial memory retrieval suggests a shift of the hippocampal spatial representations from coding the current spatial context to coding the remembered environment. In this study we show in rats that the CA3 neuronal population can switch spontaneously across representations and transiently activate another stored familiar spatial pattern without direct external sensory cuing. This phenomenon qualitatively differs from the well-described sharp wave-related pattern reactivations during immobility. Here, it occurs under the theta oscillatory state during active exploration and reflects the preceding experience of sudden environmental change. The respective out-context coding spikes appeared later in the theta cycle than the in-context ones. Finally, the experience also induced the emergence of population vectors with a co-expression of both codes segregated into different phases of the theta cycle.

## Introduction

A prerequisite of episodic memory recall is the ability to reactivate previously encoded neural activity patterns independently of the current spatial context. It is self-evident that in humans it can occur across different awake behavioral states such as immobility or active locomotion. Episodic memory in humans, spatial memory, and allocentric navigation across mammalian species critically require a functional hippocampus^1,2^. Regardless of whether or not we consider laboratory animals capable of remembering a full set of human episodic memory dimensions – semantic, spatial, temporal and emotional – the understanding of how the hippocampus encodes and how it recalls the spatial information provides important knowledge closely related to the mechanisms of episodic memory in general.

Hippocampal principal cell activity is strongly modulated by the subject’s position in space, forming context-specific neural representations that likely provide a physiological substrate of spatial memory^3^. Memory recall in general is a very volatile process between the cue appearance and the behavioral response. When thinking about an episodic memory recall, one would assume that its spatial component requires the reactivation of the respective spatial representation in the hippocampal networks, thereby shifting from the code for the subject´s actual location to the stored spatial code of the episode that might have occurred in an entirely different context. Analysis of the spiking activity from populations of individual place cells enables the identification of the evolution of the spatial information code in a high temporal resolution and to capture eventual transient shifts between in-context and out-context related hippocampal representations.

In the literature on rats and mice, there are a considerable number of studies on the reactivation of hippocampal spatial representations that were encoded in a different context than the one the animal occupies at the time. However, they were shown during sleep^4–6^ or during animal´s awake immobility^7^, in both cases when the networks expressed an ‘off-line mode’ characterized by large irregular activity (LIA) with occasional sharp wave/ripple complexes (SWR). These instances are considered part of a consolidation process rather than the element of memory recollection^8–10^. While the animal performs locomotion, theta oscillation (6–12 Hz) dominates in the hippocampal local field potential and the place cell population activity mainly codes for its actual position. Knowledge is being expanded about the ‘non-local’ patterns generated during ongoing theta. This knowledge refers to distant positions, but within the same environment context, instead reflecting planned or considered actions to be performed within the imminent future^11–14^. A spontaneous out-context spatial pattern recall that would emerge during the awake theta oscillatory state has not been described so far.

Jezek et al. (2011) showed that the hippocampal CA3 network can transiently switch from a representation reflecting the current environment to a map of an independent context and eventually flicker back and forth for couple of seconds, while being paced by the local theta oscillation. The emergence of this phenomenon immediately followed the ‘teleportation procedure’ in which the rat experienced a sudden change between the visual sensory inputs identifying the two respective familiar environments. The transient retrieval of the out-context pattern could be as short as a single theta cycle (TC) and its scattered emergence lasted only for couple of seconds after the sensory switch^13,15^. Given its short life, it is likely that it originated from the conflict between the visual inputs that responded to the changed cue conditions in the apparatus, and the self-motion input. After the path integrator reset, the network flickering stabilized into the state that corresponded to the actual environment.

In this work we searched for a retrieval of out-context place cell patterns in the hippocampal CA3 network that would (1) emerge during ongoing theta activity, and (2) occur spontaneously without being immediately preceded by any kind of external cuing. Rather, we exposed the animals to the experience of a contextual cues switch that, as shown previously, causes the shift in the respective hippocampal representations, and we searched for a spontaneous occurrence of the map shifts after a longer delay. We found spontaneous recalls of the out-context spatial CA3 patterns corresponding to the alternative contexts in sessions delayed by 20–60 min after the context-switching experience.

## Methods

### Subjects

A total of 14 male Long-Evans rats ranging from 6 to 9 months of age and weighting between 400 and 500 g were housed individually in Plexiglass cages with transparent cover with stable 12 h/12 h light/dark cycle. Experiments were performed during the light phase. During training, the animals had limited access to food and were kept around 90% of their free-feeding body weight, while water was accessible ad libitum.

All protocols followed in this study were approved by the Ethical Committee of the Ministry of Education, Youth and Sports of the Czech Republic (approval no. MSMT-10669/2016) according to the Guide for the Care and Use of Laboratory Animals (Protection of Animals from Cruelty, Act No. 246/92, Czech Republic). The study was performed in compliance with the ARRIVE guidelines^16^.

### Electrode preparation and surgery

Rats were implanted with a “hyperdrive” containing a circular bundle of 16 independently movable tetrodes. The tetrodes were twisted from four 17 μm polyimide-coated platinum-iridium wires (90% and 10%, respectively; California Fine Wire Company). Their tips were plated with platinum to reduce their impedances to 120–200 kΩ at 1 kHz.

Before surgery, the animals were deprived of food for 12 h. Anesthesia was induced by placing the animal in an enclosed box filled with isoflurane vapor and then the intraperitoneal injection of ketamine (90 mg/kg) and xylazine (10 mg/kg) was delivered. The animal was fixed into a stereotactic apparatus with continuous influx of air (1500 ml/min) containing 1.5–3% isoflurane. The Isoflurane flow was regulated depending on breathing and reflex patterns. The tetrodes were placed above CA1 of the right hippocampus at coordinates of 3.8 mm posterior and 3.00 mm right to bregma. The hyperdrive was then anchored to the skull with jeweler screws and embedded into dental acrylic. Two screws served as an electric ground. After the surgery, the rats were allowed to recover for 1 week before the training started.

### Tetrode positioning and recording procedure

Over the course of 2 weeks following the implantation, tetrodes were slowly lowered towards their intended location in CA3 of the hippocampus. Movements proceeded in steps of 50 μm or less and were halted when a large-amplitude theta-modulated complex-spike activity appeared. One tetrode was left in the corpus callosum to serve as a reference. Several hours before the experiment started, the signal was fine-tuned. The final positions of the tetrodes were checked histologically after the experiment had finished.

The hyperdrive was connected to a multichannel, impedance matching, unity gain headstage. The output of the headstage was conducted via a lightweight multiwire tether cable and 82-channel slip-ring commutator to a data acquisition system containing 64 digitally programmable amplifiers (Neuralynx, Bozeman, MT, USA). Unit activity was amplified by a factor of 3000–5000 and bandpass filtered from 600 to 6000 Hz. Spike waveforms crossing an individually set threshold (30–80 μV) were time-stamped and digitized at 32 kHz. EEG signals, 1 per tetrode, were amplified by a factor of 1000 and recorded continuously (bandpassed between 0.5 and 475 Hz) at a sampling rate of 2 kHz. Light emitting diodes (LEDs) on the headstage were used to track the animal’s position every 40 ms.

### Behavioral training procedures and experimental setup

Both experimental and control groups each contained 7 subjects. The procedure used was based on the protocol developed by Jezek et al.^13^ The training phase was designed to establish orthogonal spatial representations of two environments (identical 60 × 60 cm square arenas enclosed by 40 cm high black walls on white matte translucent plexiglass floor). Both contexts differed through the unique configuration of controllable LED lights on the walls and beneath the floor. The recording area was surrounded by black curtains to eliminate any external visual cues. The arena LEDs were the only available light sources.

In order to strengthen discrimination between both contexts, subjects received environment-specific food reinforcement. Before every trial, the arena was thoroughly cleaned with diluted detergent. Small cookie crumbs were randomly scattered throughout the arenas to motivate the animal to move. Vanilla and chocolate cereals were used in the context-specific manner, while unflavored cereals were available across both arenas. The environment / flavor combination was randomly chosen before the training started. In each environment, the proportion of flavored and unflavored crumbs was approximately 4:1. During the teleportation trials, only unflavored reinforcement was offered.

**Figure 1:**
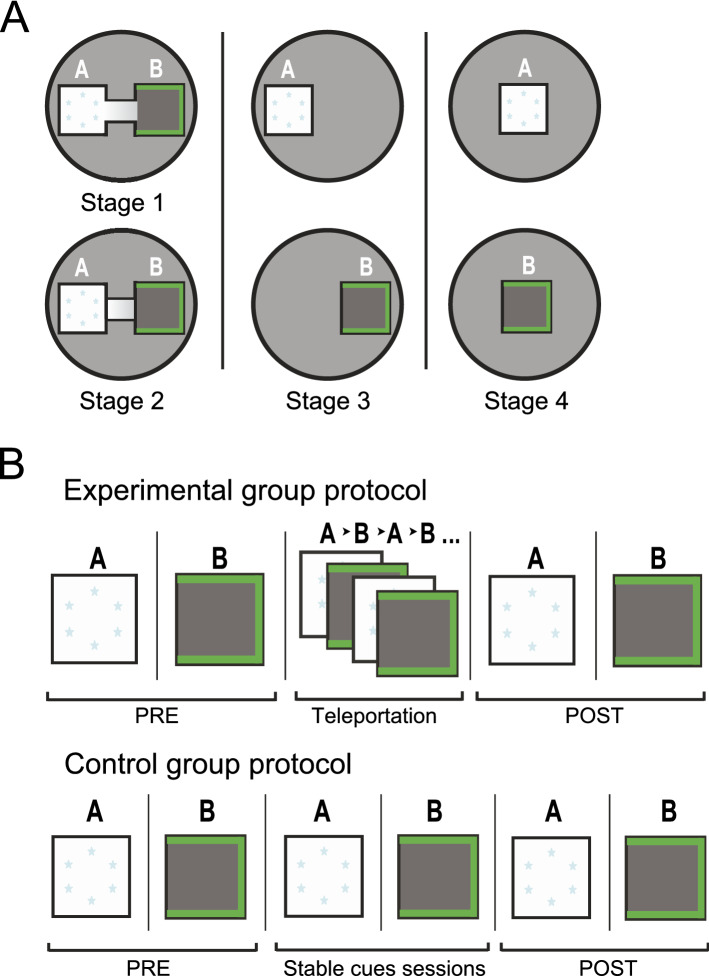
Scheme of the training and experimental paradigm. (**A**) All procedures were performed in an enclosure surrounded by black curtains. The training scheme was designed so that the animal forms two distinct spatial representations for the respective environments. (**B**) Scheme of the experimental day procedure. Initial baseline recordings (PRE) were followed with the teleportation session in the experimental group or with two sessions with steady cues in controls. After 20 and 50-min, two test sessions (POST) were recorded.

The training consisted of four stages (Fig. 1A). In the first stage (double box), arenas were located next to each other and connected by a 20 cm wide and 20 cm long passage allowing the animal to travel freely between the enclosures A and B in three 20-min sessions. In the second stage (double box disconnected), the connecting alley between the arenas was closed and the enclosures were presented separately. In the third phase (single box/two locations) we replaced the whole apparatus with a single box that was equipped with both sets of lights and presented at the original locations. Finally, in the fourth stage (single box/one location) the same single box was presented in the common location in the center of the recording chamber independently of the cues lit. Across phases 2 to 4 the rats experienced three 10-min sessions in each of the two environment identities. The order of environment presentation was randomly chosen from four predefined schemes (ABBAAB, ABABBA, BAABBA and BABAAB). Across all phases the animals rested for 20-min between the sessions in a cushioned pot on a pedestal located outside the curtains.

On the testing day (Fig. 1B), both groups were first exposed to each environment for 10 min (PRE sessions). Then, the experimental group received one teleportation session. Each rat was introduced into the arena with one set of lights on. After 60–90 s of free exploration the light cues were switched to the other set, instantaneously changing the visual discriminants of the environment. The light switching (teleportation trial) was repeated every 40–60 s for 10-min. The control group animals received two 5-min sessions, one per each environment identity spaced with 5-min break instead of the teleportation session. Afterwards, both groups again received one 10-min session in each environment under stable cue conditions (POST sessions).

### Spike sorting and cell classification

Offline spike sorting was performed manually in three-dimensional feature space depicting projections of waveform amplitudes and energies (SpikeSort 3D 2.5.1.0, Neuralynx). Only well-separated clusters were accepted into the analysis. Putative pyramidal cells were identified by their spike width, average firing rate and by distribution of their interspike intervals (occurrence of complex spike bursts). Putative interneurons were not included in the analysis. Only data from periods with movement velocity larger than 2 cm/s were included in the analysis.

### Template population vector construction

In order to define the expected spatial distribution of cell firing, the template population vectors were constructed based on the activity from PRE-sessions 1 and 2. We first split the PRE-sessions in halves and used the first part of each session to establish the activity templates, and the second half for the baseline measures. Sufficient arena coverage was required across both template periods tolerating only sparse unvisited position bins. Then, the registered track coordinates were transformed into 20 × 20 position bins (pixels) of 3 × 3 cm and the spatial ratemaps were built by dividing the number of spikes emerged in each position bin by the total time spent in it. The resulting ratemaps were smoothed using 5 × 5 Gaussian boxcar average over the surrounding bins. The weights were distributed as follows:

$$\begin{gathered} \left[ {\begin{array}{*{20}c} {0.00{25}} & {0.0{125}} & {0.0{2}00} & {0.0{125}} & {0.00{25};} \\ \end{array} } \right. \hfill \\ \begin{array}{*{20}c} {0.0{125}} & {0.0{625}} & {0.{1}000} & {0.0{625}} & {0.0{125};} \\ \end{array} \hfill \\ \begin{array}{*{20}c} {0.0{2}00} & {0.{1}000} & {0.{16}00} & {0.{1}000} & {0.0{2}00;} \\ \end{array} \hfill \\ \begin{array}{*{20}c} {0.0{125}} & {0.0{625}} & {0.{1}000} & {0.0{625}} & {0.0{125};} \\ \end{array} \hfill \\ \left. {\begin{array}{*{20}c} {0.00{25}} & {0.0{125}} & {0.0{2}00} & {0.0{125}} & {0.00{25}} \\ \end{array} } \right] \hfill \\ \end{gathered}$$

Cells that changed their spatial tuning from PRE to POST sessions (by shifting or expanding their firing field or developing a new one) were excluded from the analysis.

### Momentary population vectors building and categorization

Theta waves were identified from the local EEG using a Hamming window and a band-pass filter between 5/6 and 11/12 Hz. The cell activity-based population vectors (PV) were constructed by assigning the individual cell activity into the temporal bins defined by periods of the successive theta cycles (TC). Each temporal bin consisted of the entire theta cycle of 360°. The borders between the bins were set to theta phase with the lowest mean firing rate across the collected cell population^13^. Each population vector was linked with a corresponding position of the rat derived from the tracking file.

**Figure 2:**
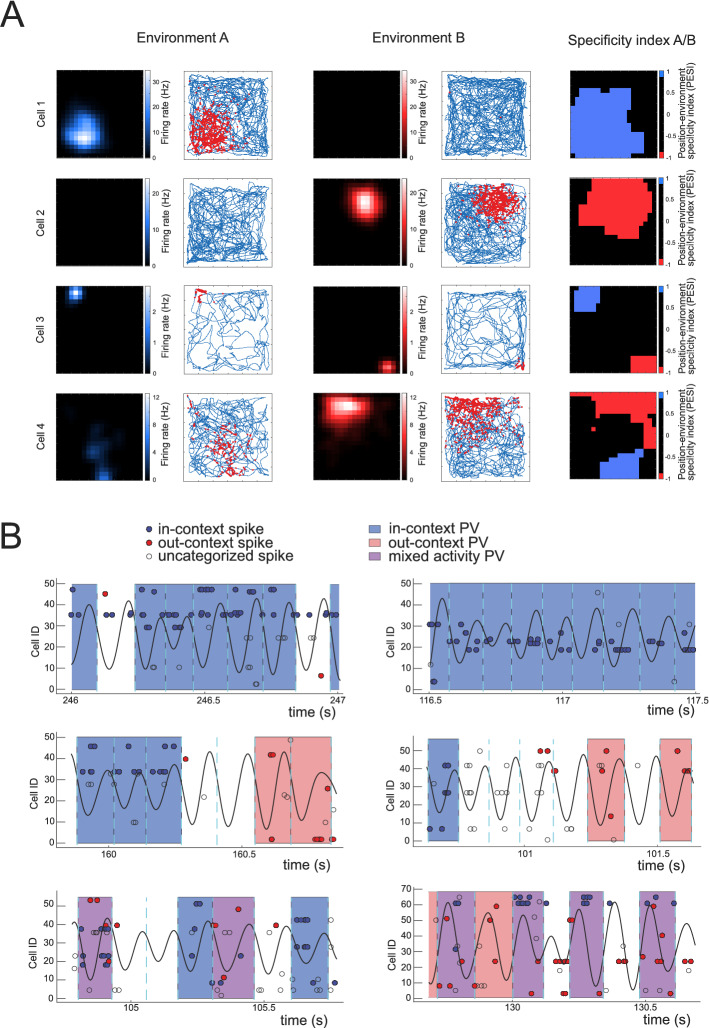
Examples of cell and population activity. (**A**) Examples of activity from four individual CA3 cells across environments A and B. The first four columns represent ratemaps and a track (blue line) with superposed spikes (red dots) from template sessions in environments A and B. The column on the right displays the position bins where the cell demonstrated the activity specific to one of the environments, either A (blue) or B (red). Some cells (# 3 and 4) were active in both environments. Only the cell´s activity in bins with the absolute specificity index was considered in later analysis. (**B**) Examples of population activity categorization. Population activity patterns categorized as in-context (ICPV) are colored with blue, the out-context (OCPV) with red, and the mixed patterns (MPV) with purple. The criteria are described in the Methods section. The black bold curve represents filtered LFP (8–12 Hz) and the dashed cyan lines show identified borders between the population vector bins. Spikes (circles) from individual cells (in lines) are marked with red or blue if they fired at the position with an absolute specificity coding, measured by the cell´s environment specificity index. The spikes that were expected to fire according to the pre-defined template in the environment present at the time, are highlighted in blue. The spikes that were expected to fire at the given coordinate only in the alternative environment are highlighted with red. Spikes categorized as nonspecific (empty circles) appeared in spatial bins without the absolute specificity for any of the environments and were disregarded from the PV analysis.

The contextual specificity of place cell activity for each position bin was assessed using ‘Position/Environment Specificity Index’ (PESI), that defined the difference between the cell’s expected activity across the environments for the given position bin. Then, for the position bin x, PESI_x_ = (f_A,x _− f_B,x_) / (f_A,x_ + f_B,x_), where f_A,x_ and f_B,x_ are the reference mean firing rates for the given bin in the respective environments. The place cell activity was considered specific in position x if the corresponding PESI_x_ had a value of − 1 or 1. The analysis was restricted only to those position bins that showed activity from at least 2 cells with PESI = 1 and another 2 cells with PESI = − 1. This ensured that we were capable of registering both context-specific patterns (A and B, respectively) for the given bin whenever they should occur. For the subsequent population vector categorization we only considered spikes from those cells that showed context-specific activity in the given position bin (Fig. 2A, Suppl. Figure 9).

Depending on the cells active in the momentary population vector, we categorized the data into ‘in-contextv, ‘out-context’ and ‘mixed’ population vectors. The first pattern, the ‘in-context population vector’ (ICPV), was identified when the given PV contained activity from two or more cells specific to the context present at the moment, and no spike from a cell specific to the alternative environment. The second pattern, the ‘out-context population vector’ (OCPV), was scored when the PV contained spikes from 2 and more cells specific to the alternative context at the given position bin, and no spikes from a cell specific to the actual current spatial context. Identification of “mixed” activity pattern (mixed population vector, MPV) required simultaneous activity of at least two cells otherwise specific for each environment in the given spatial pixel. As specified above, the only position pixels considered were those encoded by a sufficient number of specific place cells allowing to detect all activity types.

In order to control for the differences in cumulative times spent across various coordinates, we normalized the activity pattern counts. The amounts of each pattern scored in the given position bin were normalized by the total amount of temporal bins spent there. All the corresponding scores were then averaged across the whole session. They are presented in the format of the average number of activity pattern instances per 1000 theta cycles. To control for possible differences in results based on unequal cell-sample sizes across the groups, we made 10 randomly selected sub-samplings in the control group so as to equal the experimental group sample size. We then reanalyzed the incidences of various activity patterns and averaged the results of all iterations.

### Theta phase measurements

All the presented phase values are relative to theta cycle borders determined individually for each recording (0 degrees at the theta cycle border). The respective relative theta phase value was detected for each considered spike in the analyzed PV. In mixed cycles, we separately treated spikes from the place cells specific for the current and alternative environment. To perform the phase statistics, we used MATLAB Circular Statistics Toolbox (Version 1.21.0.0, Berens) using the Watson-Williams Test for equality of means with Bonferroni correction for multiple comparisons.

### Spatial and temporal properties of activity patterns

The precision of position coding (position coding error) was assessed using a correlation decoder, where the momentary population vector from the respective theta cycle was correlated with the template population vectors across all the position bins of the arena. The decoded position corresponded to the position bin with the highest correlation and the coding error was defined as the distance between the decoded and the real position of the animal. The coding error was computed for both the in-context and out-context theta cycles and averaged out across the session.

Further evaluated parameters of spatial code included: the distance from the current position to the place field center for each active cell, averaged across active cells within the theta cycle; the average distance between place field centers and proportion of active cells firing at the periphery/outside of the respective place field. The place field center was defined as the position bin with maximal reference mean firing rate. The positions at the periphery/outside of the given place field were defined as position bins with mean firing rate < 10% of the maximal rate value for the given template. For each parameter, the values were averaged across all the events of the respective activity type detected within a session. A comparison of parameters related to the positional error between the out-context vs. in-context patterns was performed using either linear mixed-effects (LME; the case of the cells firing on periphery) or generalized mixed-effects models with Gamma-errors distribution and log-link function (GLMM; all other paramters) via glmmTMB package^17^. These parameters included positional error, the number of active cells, the spatial distance of the given spike from the center of the place field, and the average distance between the place field centers. Furthermore, an out-context vs. in-context patterns comparison in terms of positional errors was also performed (using the GLMM) while adjusting for the effects of these error-related parameters, which improved model parsimony (estimated using the Bayesian Information Criterion).

We evaluated temporal relationships between the OCPV instances by measuring the average temporal distance between subsequent OCPVs and compared them to the average temporal separations measured in 5000 samples containing an equivalent number of simulated, randomly distributed OCPV instances. We then focused on OCPV/MPV clustering on a shorter timescale. We restricted the analysis to sessions containing at least 12 OCPV/MPVs. We created binary arrays, corresponding to theta binning of data. The n-th position within an array yielded 1 if the n-th theta bin expressed an OCPV/MCPV, and 0 otherwise. The dot product auto-correlations of the arrays were then computed And the auto-correlation values were then normalized by computing a z-score: z = (C-mean(|Cshuffle|))/std(|Cshuffle|), where C was the observed cross-correlation value and |Cshuffle| was the set of cross-correlation values in the shuffled data. The randomization was performed by randomly shuffling position of the OCPV/MPVs within the theta bin array. The eligible bins for OCPV/MPV replacement had to contain activity of at least two cells and occur at a rat position sufficiently covered by place fields to detect the expression of the respective spatial maps. The shuffling was repeated 1000 times for each session.

The z-scored values were used to compute the average auto-correlogram across the sessions. The significance of the OCPV/MPV cumulation at subsequent theta bins was evaluated for each analyzed session by comparing the auto-correlation value for one theta bin lag with the shuffled data. The one-sided theoretical p-value corresponds to *p* = (n + 1)/1001, where n is the number of cases in the shuffled data with value equal to or larger than in the real data.

Spatial clustering was evaluated by comparing the shortest distances among the OCPV and MPV events, respectively, with the corresponding values obtained from randomization procedures. To reliably detect instances of significant spatial clustering of OCPVs, we used two complementary randomization procedures that accounted for specific temporal structure of OCPV expression and inhomogeneities in the place field’s coverage of the arena. First, we randomly shifted the time-stamps of the OCPV events along the original track of the given session, skipping the spatial bins with place field coverage insufficient for flicker detection. Using the original track enabled us to reflect the original spatial occupancy across the arena. The average of the shortest inter-event distances was then measured. Their random distribution was obtained by repeating this step 1000 times. The OCPVs were down-sampled so that there was minimum 5 s of time and at least 20 cm of trajectory length elapsed between any two OCPV events to ensure that the spatially co-localized OCPV or MPV events occurred only when the animal had returned to the same location.

We note that while this randomization preserves the temporal structure between the original and shuffled data, it fails to precisely capture spatially non-uniform probabilities of detecting activity within a particular map, as density of place field coverage might vary considerably across the explored environment. We thus performed a complementary randomization procedure, using the other reference session (with the alternative context identity) as a control. The data were down-sampled to equalize occupancy at each location (5 × 5 bins) across both the examined and the control session. We then randomly chose an equal number of PVs expressing the same map during the control session 1000 times. The sessions with less than 5 detected OCPV events were discarded from this analysis.

### Bayesian decoding of contextual map

An alternative approach to population vector classification was based on Bayesian decoder adapted from previous work of Davidson et al. (2009)^18^. For each candidate theta cycle population vector we calculated conditional probability of the rat position at individual position bins across both environments:

$$P\left( {pos, context|spikes} \right) = \frac{{P\left( {spikes|pos, context} \right).P\left( {pos, context} \right)}}{{P\left( {spikes} \right)}}$$

We considered independent place cell firing modelled as spatially inhomogenous Poisson process defined by template firing rates, leading to:

$$P\left( {spikes|pos, context} \right) = \mathop \prod \limits_{i = 1}^{N} \frac{{\left( {t f_{i} \left( {pos, context} \right)} \right)^{{n_{i} }} }}{{n_{i} !}}{\text{exp}}\left( { - tf_{i} \left( {pos, context} \right)} \right)$$

where *t* is duration of the theta cycle, *f*_*i*_ is template mean firing rate of *i*-th neuron for given position and context and *n*_*i*_ is number of spikes fired by respective unit. The prior probabilities *P*(*pos, context*) were considered to be uniformly distributed across all the position bins.

The probability corresponding to a single environment was then equal to sum of decoded posterior probabilities for all the position bins within the environment. As a threshold for particular map detection we set *P*(*context*|*spikes*) > 0.95.

### Statistics

Pre-processed electrophysiological and behavioral data were imported into MATLAB (Version 8.4, MathWorks Inc.) and further processed using custom-written scripts. Statistical analysis was performed using MATLAB, STATISTICA (Version 12, StatSoft Inc.), Excel (Version 14.0.7214.5000, Microsoft Corporation) and R (Version 4.4.1, R Development Core Team^19^). The residuals of all models were visually checked for potential bias in model predictions and/or heteroscedasticity^20^. Data of repeated measurements with approximately homoscedastic and normally-distributed errors were analyzed using linear mixed-effects models (LME) with animal identity representing random-effect factor (random intercept), using *nlme* package^21^. The probabilities of the occurrence of specific PV patterns (ICPV, OCPV, or MPV) per single TC (possible range 0–1) were modelled using beta regression with logit link function via the *mgcv* R package^22^. For this, the probabilities of the PV patterns were averaged across both environments, separately for the PRE and POST sessions. We thus obtained 2 data points per animal and each PV pattern. Next, we performed the beta regression model, with the probability of the PV pattern as an outcome, animal identity as a random-effect factor (with random intercept) and phase (PRE vs. POST), group (experimental vs. control) and their interaction (‘phase * group’) as fixed-effects predictors. The models were parameterized in such a way that the PRE phase in a control group represented an intercept. As beta regression does not allow for the occurrence of zeros, data were bounded to ¾ of the minimal non-zero value of the given vector^17^. In order to robustly evaluate the accuracy of the estimated effects (regression coefficients [β] obtained from beta regression [in logit] and LME, fold-change/difference for Gamma GLMM), a non-parametric bias-corrected and accelerated bootstrap (5000 resamplings^23^) was used to compute 95% confidence intervals. For of mixed states incidence, we used a percentile bootstrap due to polymodal distribution of simulated estimates and thus possibly inaccurate bias correction. Whole clusters of correlated data were resampled to preserve within-subject dependency^24^. In analyses of within-session trends, the time bin was treated as a numerical variable with presumed linear effect, since its categorization increased the *Bayesian information criterion*^25^.

### Histology and electrode positions

Rats were terminated using overdose of pentobarbital and were intracardially perfused with ringer solution followed by 4% formaldehyde. The brains were extracted and stored in formaldehyde to be subsequently frozen and cut into 30 μm coronal sections and stained in cresyl violet. Tetrode tips were located by comparing protocols and tissue damage in adjacent sections (Suppl. Figure 8). Electrodes that putatively ended in CA1 or in dentate gyrus were disregarded from the data set.

## Results

**Figure 3:**
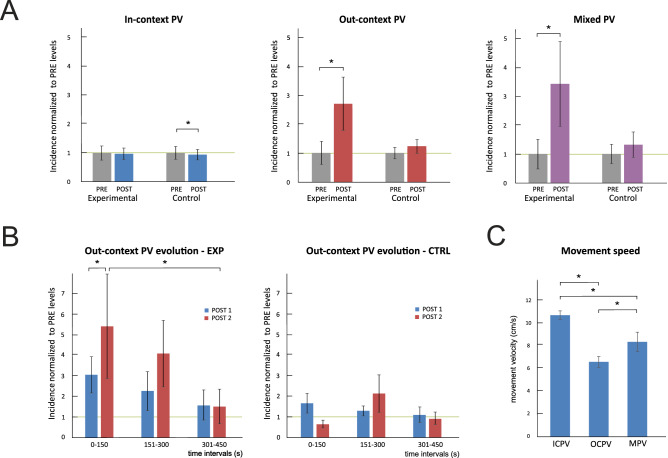
Incidence of in-context, out-context and mixed activity patterns. (**A**) Relative changes in incidence of all the categorized activity patterns across the experimental and control group. All values are normalized to the respective PRE-teleportation sessions baseline (green line). (**B**) Evolution of out-context activity patterns incidence within both POST sessions, divided into three 150-s intervals. The emergence of OCPV was high at the beginning of the session and decreased gradually in the experimental group with the teleportation session experience. (**C**) Average subject’s movement velocity during each identified activity pattern. * indicates *p* < 0.05.

We identified 585 place cells across all the subjects (mean 29.25 ± 3.25 cells per rat). For each animal, we found on average 253.00 ± 19.39 out of 400 position bins that met our criteria for environment-specificity coding and behavioral coverage (see Methods, Fig. 2B). Initially, we detected the baseline emergence of out-context population vectors during the PRE sessions. The average incidence across all animals was 1.553 ± 0.439 OCPV/1000TC. In POST sessions data we found that the experience of repetitive teleportation in the experimental group led to a significant increase in the incidence of out-context population vectors approximately 2.7 times (from 1.300 ± 0.493 OCPV/1000TC (PRE) to 3.571 ± 1.225 OCPV/1000TC (POST); β = 0.971 [0.668, 1.274], *p* < 0.0001, beta regression; Fig. 3A, Suppl. Figure 1). This contrasted with data from the control group exposed to stable environment sessions instead of teleportations that did not show any significant change in OCPV (PRE: 1.806 ± 0.384 OCPV/1000TC; POST: 2.371 ± 0.477 OCPV/1000TC; β = 0.261 [− 0.038, 0.561], *p* = 0.087, beta regression). The increase observed in the experimental was significantly higher than the change by a factor of approximately 1.3 detected in the control group (β = 0.710 [0.284, 1.136], *p* = 0.001, beta regression). The PRE OCPV incidences in experimental and control group were comparable (β = − 0.880 [− 1.961, 0.201], *p* = 0.111, beta regression).

A similar effect was observed in the mixed population vector analysis (MPV). In the experimental group, the teleportation experience led to an increase in the emergence of MPV from 0.589 ± 0,309 MPV/1000TC in the PRE sessions to 2,045 ± 0.932 MPV/1000TC in the POST sessions (β = 1.110 [0.801, 1.419], *p* < 0.0001, beta regression; Fig. 3A, Suppl. Figure 1). The control group without the teleportation experience did not show any significant increase as the resulting values were rather comparable (PRE: 0.510 ± 0.166 MPV/1000TC; POST: 0.744 ± 0.248 MPV/1000TC; β = 0.311 [− 0.082, 0.704], *p* = 0.121, beta regression). The 3.5 fold increase in MPV emergence in the experimental group was significantly higher than the 1.5-fold increase observed in the control group (β = 0.799 [0.230, 1.299], *p* = 0.002, beta regression).

In the next step we analyzed the evolution of OCPV emergence within the sessions. All PRE and POST sessions were divided into thirds and the corresponding OCPV events were counted. We found a stabilization effect within the POST sessions in the experimental group data. The average incidence of OCPV decreased between the session’s first and last third from 5.094 ± 1.632 OCPV/1000TC to 1.775 ± 0.706 OCPV/1000TC when both POST sessions were pooled (Fig. 3B; ẞ (1 time bin) =  − 0.496 [− 0.739, − 0.254], *p* < 0.0001, beta regression). When analyzing the data from the POST1 and POST2 sessions individually, we found a similar trend, but which only reached significance in the POST2 (POST1: from 3.636 ± 1.003 OCPV/1000TC to 1.856 ± 0.959 OCPV/1000TC, β = − 0.255 [− 0.578, 0.067], *p* = 0.121, beta regression; POST2: from 6.551 ± 3.080 OCPV/1000TC to 1.694 ± 1.089 OCPV/1000TC, β = − 0.591[− 0.874, − 0.307], *p* < 0.0001, beta regression). There was a significantly higher amount of OCPV events in the POST2 session than in the preceding POST1 session (β = 0.698 [0.208, 0.067], *p* < 0.01, beta regression). The fact that the effect was stronger in the second POST session was rather surprising and suggests that the network stabilization after a conflicting sensory experience has a long-term non-linear pattern. No such effects of changing instability were observed in the control group (POST from 2.134 ± 0.543 OCPV/1000TC to 1.712 ± 1.528 OCPV/1000TC, β = 0.068 [− 0.142, 0.277], *p* = 0.528, beta regression, subsampled to exp. group PV size). None of the PRE sessions in either the experimental or control group demonstrated such evolution in within-session OCPV presence (EXP: from 1.111 ± 0.203 OCPV/1000TC to 1.178 ± 0.215 OCPV/1000TC, β = 0.038 [− 0.236, 0.312], *p* = 0.785, beta regression; CTRL: from 1.672 ± 0.379 OCPV/1000TC to 1.864 ± 0.417 OCPV/1000TC, β = − 0.008 [− 0.195, 0.179], *p* = 0.933, beta regression, normalized PV size). The average MPV scores in the experimental group changed from 2.515 ± 1.251 MPV/1000TC to 1.280 ± 0.623 MPV/1000TC during the POST sessions, but their low individual counts could not be statistically evaluated.

Did the animals behave differently in relation to the categorized population vector types? We found that the OCPV events were associated with slower average speed than the ICPV and MPV categories, respectively (ICPV:10.72 ± 0.40 cm/s; OCPV 6.59 ± 0.48 cm/s; MPV 8.27 ± 0.88 cm/s). The differences in movement speed were significant between all combinations of activity patterns (Fig. 3C; ICPV × OCPV: β = − 3.918 [− 4.561, − 3.098], *p* < 0.001; ICPV × MPV: β = − 1.778 [− 2.809, − 0.930], *p* < 0.001; OCPV × MPV: β = 2.140 [0.999, 3.103], *p* < 0.001, all were LME, corrected for multiple testing). Comparison of average velocities during entire sessions did not return significant difference across the PRE and POST trials neither in the experimental nor the control group. In the experimental group, the average PRE speed was 10.45 ± 0.35 cm/s and the POST speed was 10.76 ± 0.35 cm/s (β = − 0.306 [− 0.820, 0.207], *p* = 0.257, LME). In the control group, it was 9.32 ± 0.59 cm/s in PRE and 9.23 ± 0.61 cm/s in POST (β = 0.089 [− 0.424, 0.603], *p* = 0.737, LME). Between the groups, there were comparable movement velocities in both phases before (β = 1.129 [− 0.729, 2.970], *p* = 0.268, LME) and after the teleportation session (β = 1.525 [− 0.415, 3.465], *p* = 0.140, LME). In respect to the decreasing rate of OCPV emergence within the POST sessions in the experimental group, we also analyzed evolution of animal velocity within the sessions. We detected significant decrease in velocity from the first to the last third for all PRE and POST sessions irrespectively of the group (analysis and plots in Suppl. Figure 5). The distribution of momentary speed values related to individual population vectors did not differ across the groups.

**Figure 4:**
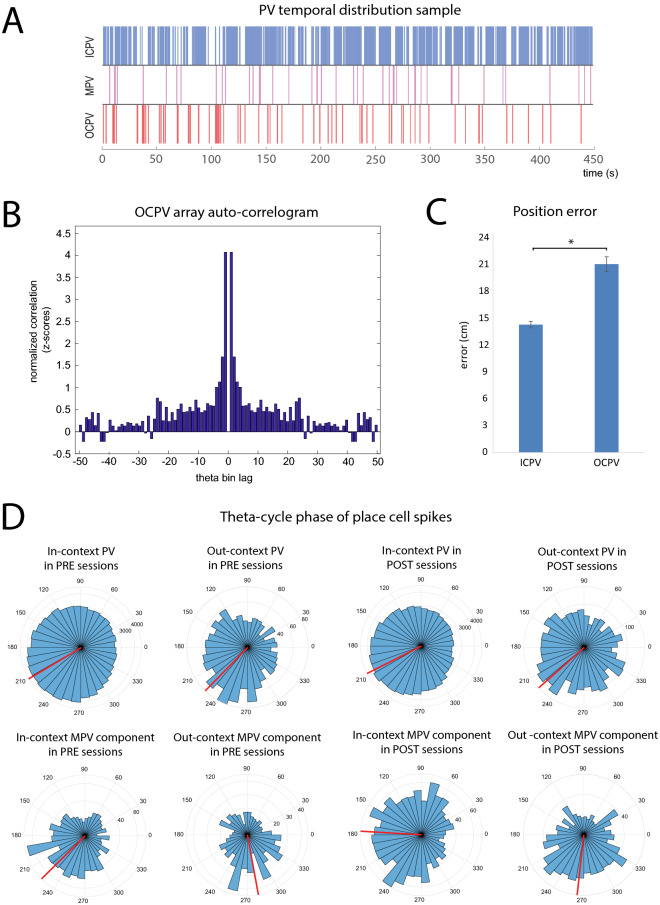
Properties of the identified activity patterns. (**A**) Example of temporal distribution of classified activity patterns during one of the POST sessions from an experimental group animal. Top: out-context PV, middle: mixed PV, bottom: in-context PV. le MPV and red OCPV; vertical separation of activity types serves for better visual differentiation of activity instances. (**B**) Out-context PV incidence auto-correlogram. The values were z-scored using random distribution and then averaged across sessions. (**C**) Deviation between the decoded and actual position of the animal across OCPV and ICPV. The out-context PV events demonstrated higher position error than the ICPV events. (**D**) Distribution (blue) and average (red) theta phase of spikes from identified in-context, out-context, and mixed population vectors. The bar length represents the number of PVs in the respective phase interval. Phase values are normalized to the individual identified phase border between the theta bins (see the Methods section). Spikes from OCPV appeared in significantly later phases of the theta cycle than ICPV. The in-context and out-context activity components of the mixed PVs appeared in significantly different portions of the theta cycle.

In the next steps we evaluated whether the OCPV expression followed any temporal or spatial pattern. The mean interval between the two subsequent OCPV events was significantly shorter than their random distribution across the whole session (data: 46.511 ± 4.923 s; random: 71.018 ± 7.020 s; U(n_1_ = n_2_ = 63) = 3487, z = − 2.503, *p* = 0.012). Given that this difference might not only point to the within-session re-stabilization effect shown above, we also focused closely on the short inter-event intervals. This analysis was restricted to a subset of sessions (n = 21) showing at least 12 OCPV occurrences. We constructed binary PV arrays mapping the emergence of OCPV across the given sessions (Fig. 4A), and calculated their autocorrelation. We found a considerable cumulation of short lag values peaking at 1 theta bin lag (Fig. 4B; one-sided *p*-values: *p* < 0.05 in 17/26 sessions). This suggests that while the majority of detected OCPV occurred in temporal isolation (Suppl. Figure 3), there was relatively frequent emergence of out-context patterns within subsequent theta cycles. A similar tendency was apparent in the case of MPV (one-sided *p*-values: *p* < 0.05 in 6/12 sessions, Suppl. Figure 4). We further evaluated the spatial distribution of OCPV pattern emergence. We considered any spatial clustering pattern as reliable only if significance had been detected using both of the approaches described in the method section. This was the case only in 1 out of the 21 analyzed sessions. We thus conclude that there was no reliable evidence for OCPV to be linked to specific coordinates and that they rather occur at various locations throughout the explored environment.

In order to evaluate the quality of spatial code across the ICPV and OCPV events, we applied a spatial position decoder. The positional error was 1.442-fold higher (95% CI 1.327, 1.567) in OCPVs than in their corresponding control in-context states (Fig. 4C; ICPV: 14.295 ± 0.366 cm, OCPV: 21.063 ± 0.822 cm, *p* < 0.001, Gamma GLMM). To further assess the OCPV associated spatial activity, we compared the ICPV and OCPV data through various other related parameters (Suppl. Figure 7). Spikes detected during OCPVs were associated with 1.302 (1.223, 1.387)-fold higher average distance from the respective cells’ place field center (*p* < 0.001, Gamma GLMM). This was reflected by higher proportion of OCPV associated cells´ firing at periphery of their firing fields (by 18%, β = 0.183 [0.139, 0.226], *p* < 0.001, LME) and by 1.209-fold higher (1.093, 1.336) average distance between centers of cells´ place fields (*p* < 0.001, Gamma GLMM) than in ICPVs. On the other hand, the number of active place cells per theta cycle was not significantly different between ICPV and OCPV (1.014-fold [0.969, 1.060] higher for OCPV, *p* = 0.5, Gamma GLMM). As a complementary analysis we performed Bayesian decoding of contextual representation for individual theta cycles. We detected increase in out-context pattern frequency from PRE to POST in experimental (PRE: 7.455 ± 2.058 PV/1000, POST: 16.771 ± 5.118 PV/1000 TC; TC β = 0.780 [0.485, 1.075], *p* < 0.0001) and control groups (PRE: 17.003 ± 5.530 PV/1000, POST: 21.941 ± 4.097 PV/1000 TC; TC β = 0.270 [0.044, 0.496], *p* = 0.019) . The increase in the experimental group was significantly higher than increase in the control group (β = − 0.510 [− 0.881, − 0.139], *p* = 0.007). This further supports the notion that teleportation experience facilitates expression of out-context memory pattern. Furthermore, the Bayesian information criterion (BIC) suggested that in addition to the PV type (ICPV vs. OCPV), inclusion of the above-mentioned parameters (except for the number of active place cells) markedly improved the prediction of the positional error. The best prediction was achieved after the inclusion of the distance from the place field center, leading to decrease in BIC by 41. After the adjustment of this predictor, OCPV still showed noticeably higher positional error (1.224-fold [1.122–1.337], *p* < 0.001, Gamma GLMM) although the estimated difference was smaller. This indicates that the OCPV were associated with increased activation of place cells at locations associated with relatively low probability of firing, which could contribute to their compromised spatial coding.

## Theta related properties

The frequency of theta rhythm during the different activity patterns did not follow the observed velocity differences. The average theta rhythm frequency of 8.205 ± 0.058 Hz during ICPV did not differ from the average frequency during OCPV (8.416 ± 0.176 Hz; β = 0.027 [− 0.007, 0.060], *p* = 0.355) or during MPV (8.008 ± 0.335 Hz; β = − 0.023 [− 0.062, 0.016], *p* = 0.759). The only statistically significant difference was between OCPV and MPV events (β = 0.050 [0.009, 0.090], *p* = 0.049; Suppl. Figure 6). The categorized patterns did not differ in their momentary power of theta frequency band relative to the rest of the oscillatory spectrum (ICPV 13.204 ± 0.443, OCPV 12.279 ± 0.462, MPV 12.032 ± 0.725; F(2, 186) = 1.49, *p* = 0.228).

We then analyzed the theta phase locking for spikes occurring across all population vector categories. The respective phase values for ICPV spikes (209.105 ± 0.153°) were significantly preceding the spike phases of the OCPV events (223.733 ± 0.972°; F(1, 238,067) = 61.596, *p* < 0.0001; Suppl. Figure 2B). Because some OCPV events were identified also during the PRE sessions, we asked whether the phase shift between ICPV and OCPV differed in sessions preceding and following the teleportation experience. Within the experimental group, we found literally the same phase difference in data recorded before (PRE- ICPV: 212.908 ± 0.153°, OCPV: 223.697 ± 0.949°; F(1, 121,791) = 15.434, *p* < 0.001) and after (POST- ICPV: 205.320 ± 0.152°, OCPV: 223.763 ± 0.984°; F(1, 116,274) = 52.534, *p* < 0.001) the teleportation session (Fig. 4D). This suggests that the spontaneous and teleportation-experience induced OCPV events likely represent the same phenomenon.

Regarding mixed population vector data, we split the corresponding spikes into in-context and out-context activity components (in relation to the actually present environment) based on the same criteria used for the PV assignment. In-context and out-context components of mixed PVs showed considerable phase shift both in PRE (in-context: 227.611 ± 1.203°, out-context: 278.010 ± 1.492°, F(1, 2329) = 72.602, *p* < 0.001) and POST sessions (in-context: 176.445 ± 1.258°, out-context: 264.754 ± 1.456°, F(1, 2743) = 330.251, *p* < 0.001; Fig. 4D). The shift between the in-context (203.237 ± 1.247°;) and out-context (269.154 ± 1.471°) components of the mixed PVs was also confirmed for combined PRE and POST sessions (F(1, 4589) = 312.915, *p* < 0.001; Suppl. Figure 2B).

To deal with the unequal number of categorized theta cycles in different animals, we confirmed the phase shift effects by comparing the respective average values obtained across the individual PRE and POST sessions (Suppl. Figure 2). We found that the OCPV related spikes occurred on average 24.365° later than those from the ICPV data (ICPV: 206.936 ± 4.236°, OCPV: 231.301 ± 6.961°; F(1,149) = 9.358, *p* = 0.019). For MPV events, the in-context spikes preceded the out-context spikes by a phase shift of 92.300° within the given theta wave (in-context: 208.435 ± 9.23°, out-context: 300.735 ± 9.431°, F(1, 80) = 28.164, *p* < 0.001; Suppl. Figure 2).

## Discussion

In this paper, we described the occurrence of spontaneous transient recall of hippocampal CA3 place cell patterns referring to a context that was different than the one the subject was currently actively exploring. We showed that the emergence of out-context patterns increased after the experience of repetitive sudden contextual changes delivered by the teleportation procedure^13^ and it tended to stabilize across the post-teleportation stable-cues sessions. We found that spikes of the two pattern classes (the in-context and the out-context) showed a different theta phase modulation. The cell activity coding the regular in-context pattern appeared earlier within the theta cycle than those from the pattern referring to the alternative environment context.

In recent years and decades, various forms of non-local coding have been described under different conditions – some refer to more or less distant positions within the actually present context^11,12,14^, while others to an entirely different one. The expression of the population code referring to the true remote context has been iconically connected to the neural pattern replays during sharp wave/ripple events. This phenomenon is considered to be part of memory consolidation^8–10^. Its occurrence is usually observed in a resting box, where the animal dwells immobile or is sleeping right after the experience in the experimental apparatus, or during transient immobility epochs of active exploration^7^. In such situations the hippocampus expresses large irregular activity dominated by slow waves and the circuit is to a large extent disconnected from sensory information flow as if it was in an ´off-line mode´.

In contrast, during active exploration the hippocampal local field potential is dominated by theta oscillation (8–12 Hz) and the populations of place cells mostly code for the animal´s actual position. In addition to this, there is growing body of evidence showing that the considered or planned actions are also reflected during the theta state, in the form of activating the place cell patterns that correspond to more or less distant locations or position code sweeps within the present context. Hippocampal place cell sweeps were initially described in the vicarious trial and error paradigm (VTE)^14^, when the rat was preparing for the left or right turn response at the choice point of the continuous T-maze. While ongoing theta rhythm, subsets of place cells that corresponded to the available routes from the choice point were activated in a sequential manner, ´modelling´ the future responses in a time-compressed manner. More recently, constantly present theta sequences^26^ were identified as the animal was either actively exploring the environment or preparing for a behavioral choice. Both in the cases of VTE and various theta sequences observations the animal generated ´mental sweeps´ into possible locations within the same environment context that might represent a neuronal substrate of planning the immediate future responses.

In contrast, this report shows the emergence of patterns related to an entirely different context while exploring the environment under the ongoing theta oscillation. The described spatial representation shifts were inserted within periods of the actual position coding, and were rather sparse and short, ranging from a single to several theta cycles. They resemble the theta paced flickering described in^13,15^ in response to sudden contextual change induced by the teleportation protocol. However, the theta paced flickering phenomenon immediately followed the substantial sensory change in form of contextual shift and lasted only for several seconds before the network stabilized in the activity state corresponding to the newly present context. This nature suggests the flickering^13^ might rather be the result of competition between the idiothetic and allothetic information inputs (path integrator vs. visual cues) resulting in rapid shifts between the respective attractor states^27^ or a short-term plasticity^28^. The instances of activating the out-context pattern here, however, occur under substantially different conditions. They occur during the sessions with the steady context present, dozens of minutes after the last teleportation event. Such conditions make the possibility that the out-context pattern expression rises from the conflict between the hippocampal inputs as rather unlikely.

In terms of attractor network theories the hippocampal representations of space behave as continuous attractor states of neural activity, tolerating subtle deviations of their inputs from the stored activity patterns^29–32^. Whereas in the case of sweeps and theta sequences the non-local population activity travels within the same continuous attractor state, the phenomenon presented here showed transient network activity shifts into a different manifold in the absence of any related sensory stimulus. This suggests that their emergence was driven intrinsically. While their amount was minimal in the first sessions, the teleportation experience led to their significant increase during the sessions that followed. However, the experimental evidence that would point to a mechanism leading to a sensory-independent shift between the representations is missing. A possible explanation could be that the preceding sequence of teleportation events with the respective representation shifts brought both representations into a temporal proximity that might reinforce an associative binding between the two. Such a link then might have increased the probability of a spontaneous retrieval of the alternative context pattern during the sessions that followed.

However, this interpretation somewhat contrasts with another interesting observation, which is the progressive reduction of the out-context pattern events rates during both POST stable-context sessions. This state-change dynamics was indeed induced by the prior teleportation procedure, as we did not see any such development in the control group. Owing to the seen progression, the out-context pattern recall was rather temporary and tended to settle down as the session was going on. It is worth noting that this stabilization effect did reset after the first post-teleportation session because it replicated itself in the following one with comparable magnitude. This suggests that a factor of expectancy of another teleportation trial might contribute to the increased rate of out-context patters activation at the beginning of each post-teleportation session.

We also noted spontaneous appearances of mixed patterns between the representations for both contexts within the individual theta cycle temporal bins. Again, this observation was analogous to what has been described immediately after the teleportation trials during the supposed conflict between the path integration and visual information inputs^15^. Here, however, the mixed patterns emerged during stable context cue sessions taking place long time after the teleportation session, and in a limited amount even before it. Moreover, we observed a phase locking structure between the spikes specific to the present and alternative context within the mixed PV bins. The cells firing specifically in the currently present environment tended to appear earlier in the theta cycle compared to cells that were active exclusively in the alternative context. This within-theta organization resembles recently reported constant theta based referencing to possible futures in the hippocampus^12^ where the neural activity alternates between the code for present location and the possible future path. That phenomenon shows similar phase locking properties with the mixed activity introduced in this report, given that in Kay et al. (2020) the information about the present location also preceded the estimated future within the given theta cycle. From this point of view, the ‘mixed patterns’ might reflect referencing to the alternative environment context rather than the non-organized mixture of the two context representations, because the out-context part of the code tended to follow after the in-context portion of the mixed pattern.

How is the lower running speed related to the expression of context-independent code? The out-context coding by its nature requires substantial sensory decoupling. While walking slower, the animals are less engaged with various sensory stimuli and we can only speculate whether under these conditions the hippocampal circuitry is more prone for spontaneous sensory decoupling.

The phenomenon of out-context spatial coding is experience-driven, as the instances of the out-context retrieval were more frequent after the repetitive teleportation protocol that suddenly changed the context identity and induced the respective representation shift. This increase was rather temporary, and the network tended to stabilize within the course of behavioral sessions. In this respect, the out-context pattern retrieval shares key features with the concept of episodic memory retrieval, namely by its context-independency, by its spontaneous and transient occurrence and by being experience-driven. The existence of episodic memory in laboratory animals is a matter of long-term debate stimulated by numerous experiments in rodents or other species^33–35^. We speculate that the out-context pattern emergence might offer a valuable model for network mechanisms of episodic memory recall. The transitory and spontaneous nature of out-context and mixed PV emergence also points towards the instability of the network activity states that represent a broadly discussed substrate of certain psychiatric disorders, namely manic disorder and schizophrenia. In this relation the reactivation of context-unrelated network activity patterns might account for the emergence of positive symptoms such as delusions or hallucinations^36,37^.

## Supplementary Information

Supplementary Information.

## Data Availability

The dataset used in this publication is available on request at karel.jezek@lfp.cuni.cz.
