# Supplementary material for: Context-independent expression of spatial code in hippocampus
Source: Sci Rep. 2022 Dec 1;12:20711. doi: 10.1038/s41598-022-25006-7 (PMC9715626; doi:10.1038/s41598-022-25006-7)
Supplement: Supplementary file 1 — Supplementary Information. [file 41598_2022_25006_MOESM1_ESM.pdf]

Context-independent expression of spatial code in hippocampus

Kapl, S., Tichanek F., Zitricky, F. & Jezek, K.

Faculty of Medicine in Pilsen, Charles University, Pilsen, 32300 Czech Republic
*correspondence should be sent to karel.jezek@lfp.cuni.cz

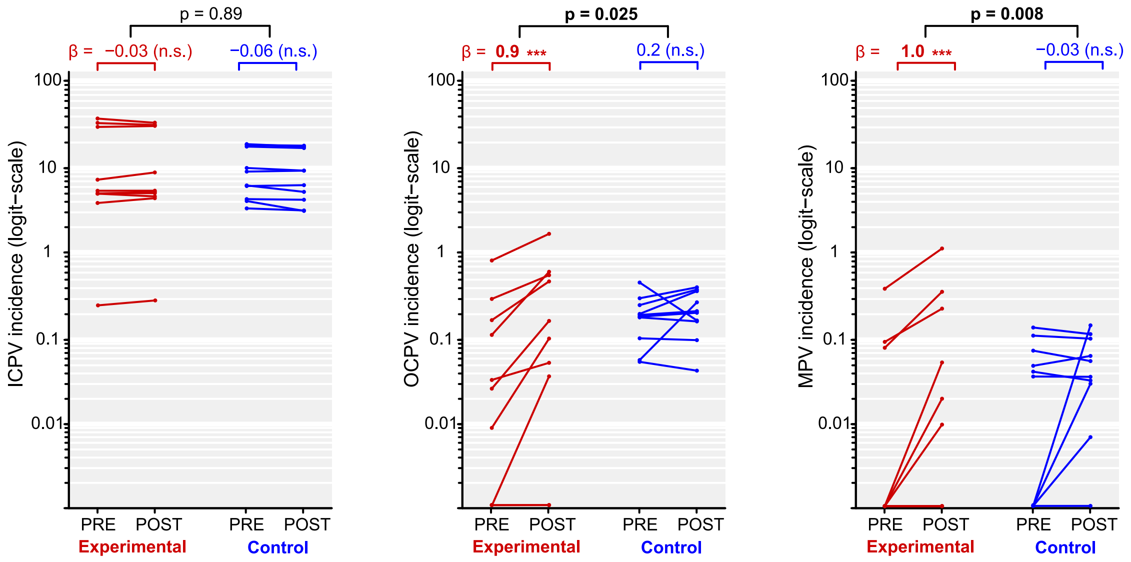

Supplementary figure 1. Changes in incidence of the categorized network activity patterns across individual experimental days.
Left panel: in-context PV; middle panel: out-context PV; right panel: mixed PVs.

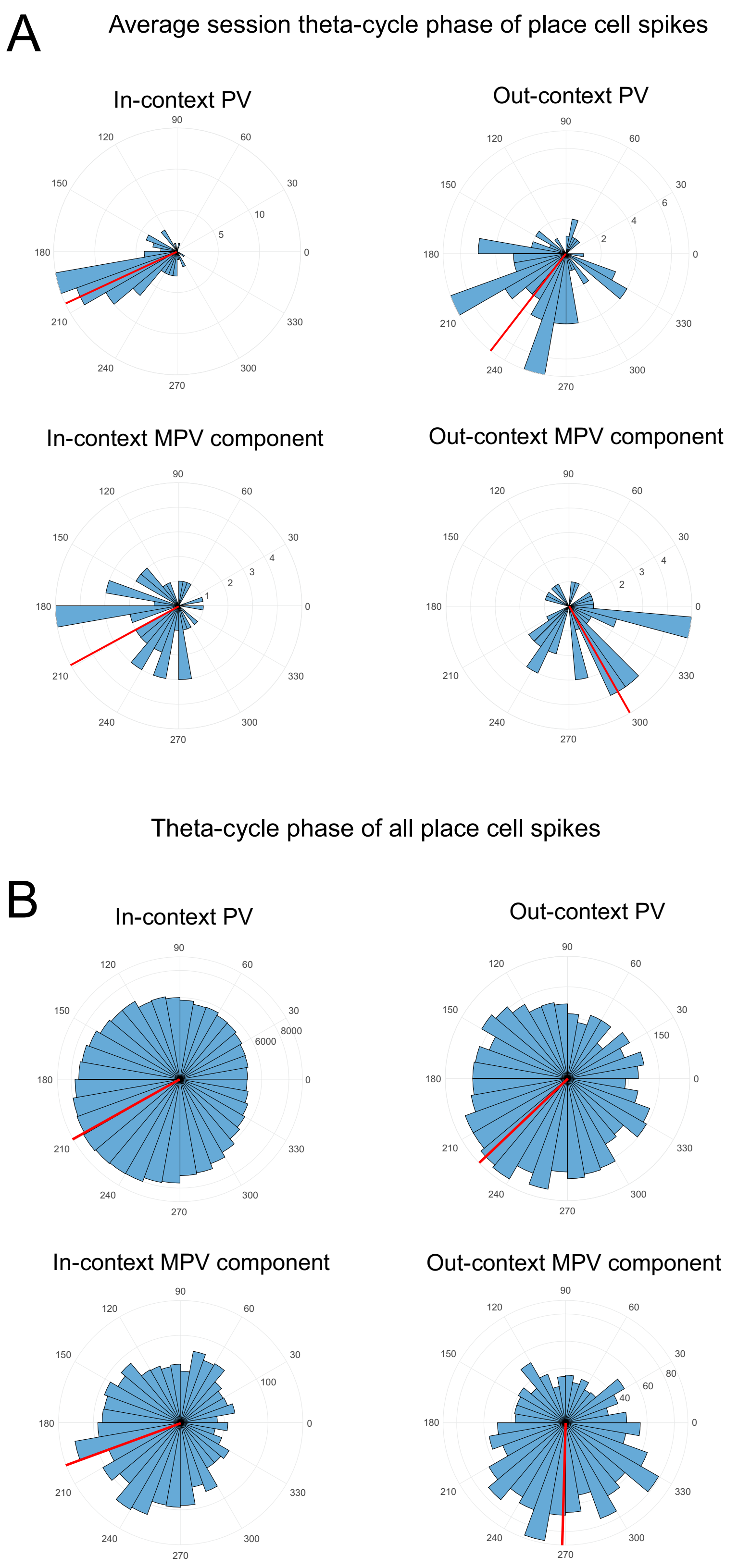

Supplementary figure 2. Distribution of spike theta phase locking.

(A) Distribution of average theta phase locking across individual sessions. (B) Distribution of individual spikes' theta phase locking across all subjects. In each figure, top left: in-context PV, top right: out-context PV, bottom left: in-context spikes from mixed PVs, bottom right: out-context spikes from MPVs. Phase values are normalized to subject-specific phase border between theta bins (see the Methods section).

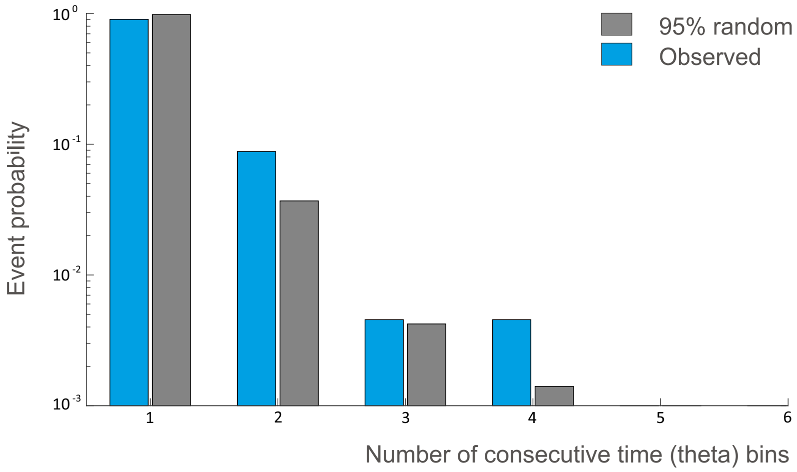

Supplementary figure 3. Duration of recalled out-context population activity patterns.

Blue bars indicate the count of all identified out-context events according their duration measured by amount of consecutive categorized bins. The grey bars show the 95th percentile of shuffled distributions, generated by 1000 times randomly choosing equal number of ICPV. The observed amounts of out-context events longer than 2 and more theta cycle bins significantly exceeded their expected random occurence.

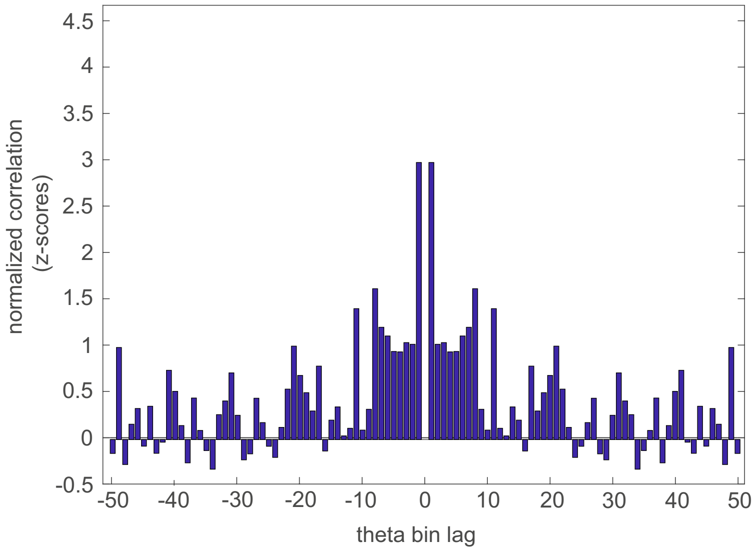

Supplementary figure 4. MPV incidence autocorrelogram.

The autocorrelation values were z-scored with respect to random distribution for each session and then averaged across sessions.

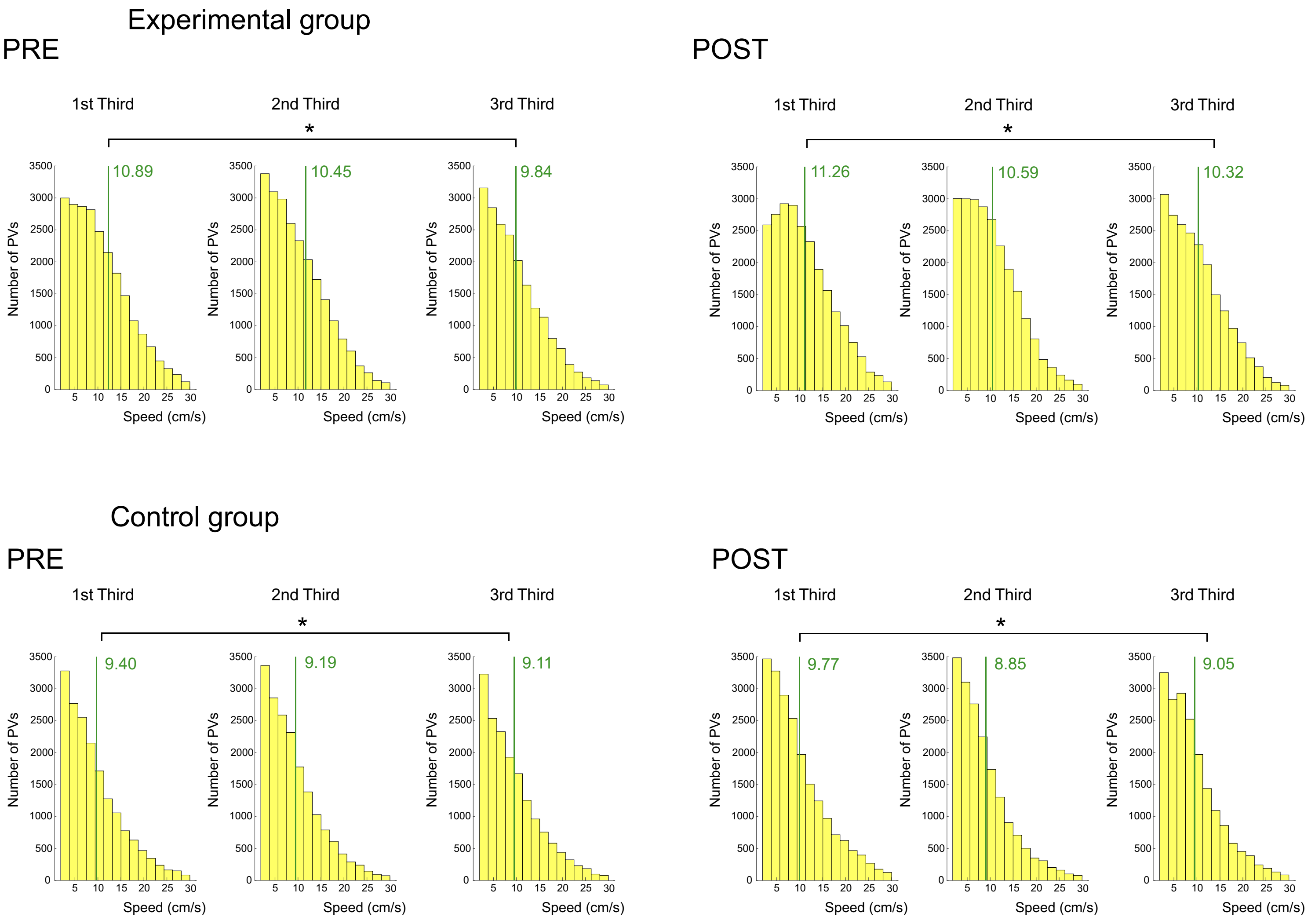

Supplementary figure 5. Distribution of movement velocity.

Distribution of subjects' movement speeds in PRE and POST sessions. Green lines represent average values. Movement velocity decreased significantly across both PRE sessions (experimental: from 10.89 ± 0.38 cm/s to 9.84 ± 0.39 cm/s, ß = -0.465 [-0.758, -0.172], p < .001, LME; control: from 9.40 ± 0.62 cm/s to 9.11 ± 0.61 cm/s, ß= -0.357 [-0.650, -0.064], p = .024, LME; experimental vs control: ß = -0.107 [-0.522, 0.307], p = .687, LME) as well as POST sessions (experimental: from 11.26 ± 1.97 cm/s to 10.32 ± 1.46 cm/s, ß = -0.465 [-0.758, -0.172], p = .004, LME; control: from 9.77 ± 2.98 cm/s to 9.05 ± 2.56 cm/s, ß = -0.357 [-0.650, -0.064], p = .022, LME) irrespectively of the group (ß = -0.107 [-0.522, 0.307], p = .615, LME).

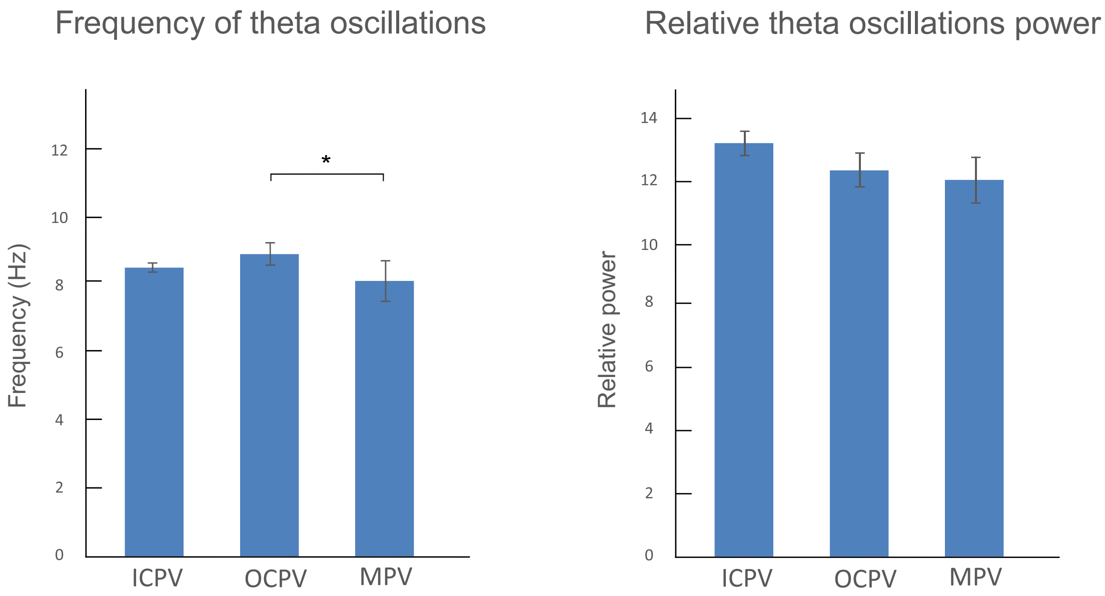

Supplementary figure 6. Frequency and relative momentary power of theta oscillations during different activity patterns.

Average frequency (left) and relative power (right) of theta oscillations during each observed activity pattern. * indicates $p<0.05$.

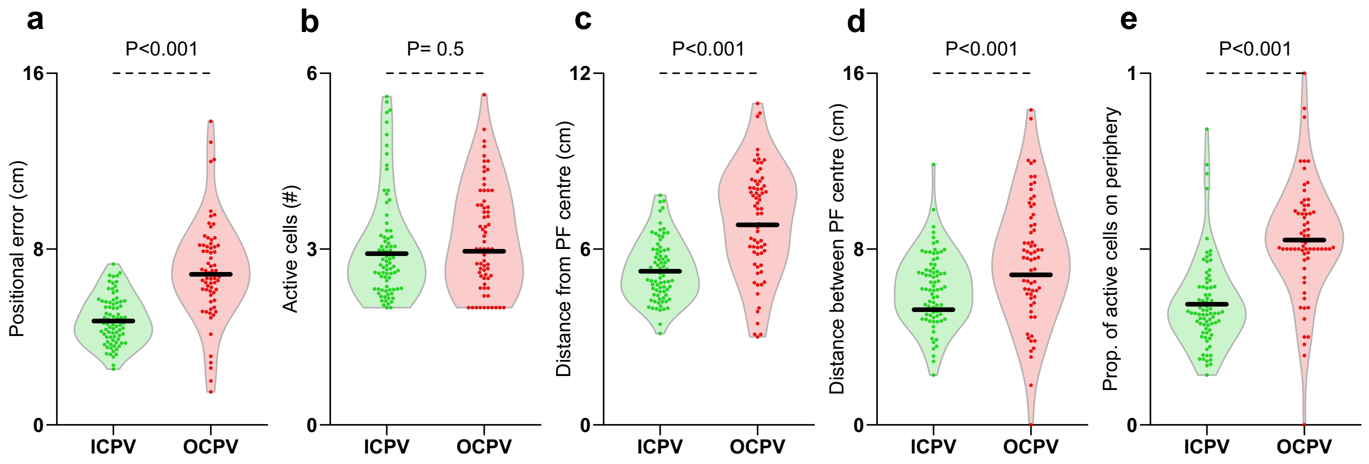

Supplementary figure 7. Spatial aspects of ICPV and OCPV.

Violin plots comparing ICPV vs. OCPV in terms of (a) positional coding errors, (b) the number of active cells per PV, (c) actual distance from centre of place field, (d) the average distance between centers of active cells´ place fields and (e) the proportion of cells firing on the periphery or out of the original place field. Thick black lines imply an estimate from model (a-d: generalized mixed-effects model with Gamma distribution, e: Gaussian mixed-effects model).

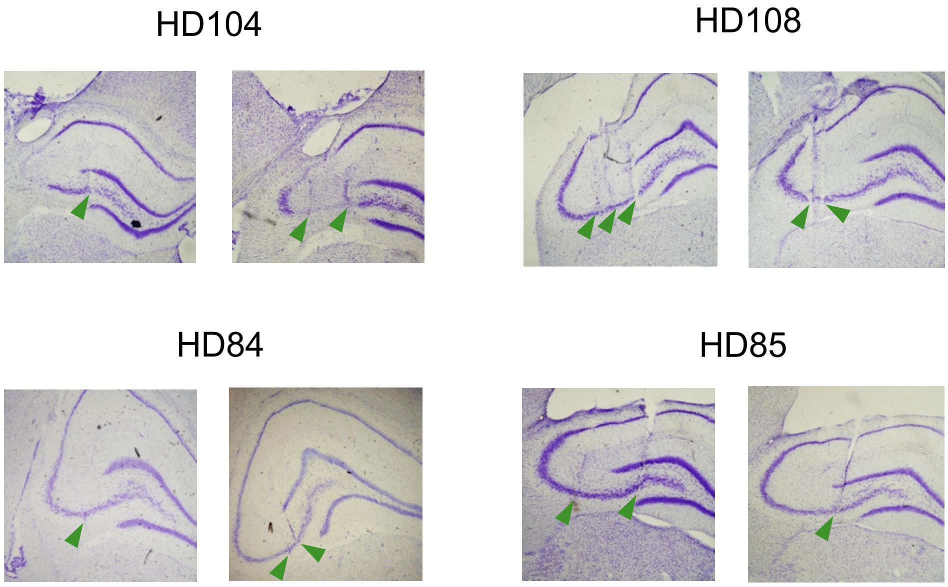

Supplementary figure 8. Histology examples with tetrode tip locations.

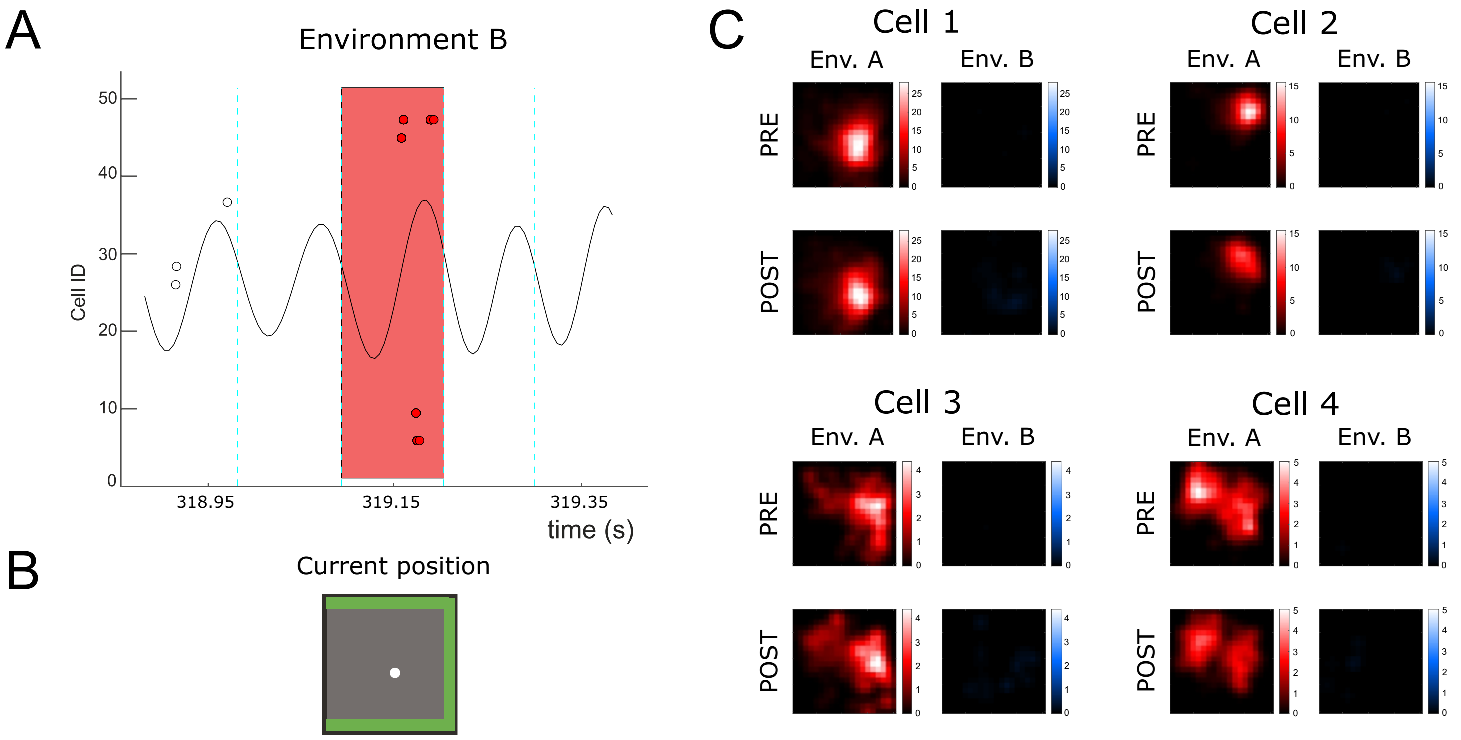

Supplementary figure 9. Cells active during an OCPV event.

(A) Sample of OCPV containing spikes from 4 place cells. (B) Subject´s position in the environment at the time shown OCPV occurence. (C) Firing fields of involved place cells in both environments during PRE and POST sessions, respectively. Blue color shows activity that is in-context for the current environment. Red color represents activity otherwise specific for the alternative environment.
